# Demographic factors in hip fracture incidence and mortality rates in California, 2000–2011

**DOI:** 10.1186/s13018-015-0332-3

**Published:** 2016-01-08

**Authors:** Kristynn J. Sullivan, Lisa E. Husak, Maria Altebarmakian, W. Timothy Brox

**Affiliations:** Psychological Science, UC Merced, 5200 Lake Road, Merced, CA 95343 USA; Orthopaedic Surgery, UCSF-Fresno, 155 N Fresno Street, Fresno, CA 93701 USA; Orthopaedic Surgery, Kaiser Permanente Medical Center, 7300 N Fresno Street, Fresno, CA 93720 USA

**Keywords:** Hip fractures, Demographics, Incidence, Mortality, Age, Gender, Race/ethnicity

## Abstract

**Background:**

Hip fractures result in both health and cost burdens from a public health perspective and have a major impact on the health care system in the USA. The purpose was to examine whether there were systematic differences in hip fracture incidence and 30-, 90-, and 365-day mortality after hip fracture in the California population as a function of age, gender, and race/ethnicity from 2000–2011.

**Methods:**

This was a population-based study from 2000 to 2011 using data from the California Office of Statewide Health and Planning and Development (OSHPD, *N* = 317,677), California State Death Statistical Master File records (*N* = 224,899), and the US Census 2000 and 2010. There were a total of 317,677 hospital admissions for hip fractures over the 12-year span and 24,899 deaths following hip fractures. All participants without linkage (substituted for social security) numbers were excluded from mortality rate calculations. Variation in incidence and mortality rates across time, gender, race/ethnicity, and age were assessed using Poisson regression models. Odds ratio and 95 % confidence intervals are provided.

**Results:**

The incidence rate of hip fractures decreased between 2000 and 2011 (odds ratio (OR) = 0.98, 95 % confidence interval (CI) 0.98, 0.98). Mortality rates also decreased over time. There were gender, race/ethnicity, and age group differences in both incidence and mortality rates.

**Conclusions:**

Males were half as likely to sustain a hip fracture, but their mortality within a year of the procedure is almost twice the rate than women. As age increased, the prevalence of hip fracture increased dramatically, but mortality did not increase as steeply. Caucasians were more likely to sustain a hip fracture and to die within 1 year after a hip fracture. The disparities in subpopulations will allow for targeted population interventions and opportunities for further research.

## Background

Hip fractures have a major impact on the health care system in the USA with an estimated incidence of 340,000 fractures annually [1]. The annual economic burden of managing hip fractures was estimated at $17–20 billion in 2010 [1, 2]. As people are expected to live longer, hip fractures will become more common. It is estimated that by the year 2050 worldwide, there will be an estimated 6.3 million hip fractures worldwide [3]. Hip fractures are a serious and life-changing event for an older person. Often after an initial hip fracture, a person cannot continue living independently and must undergo drastic lifestyle changes [4, 5]. In addition, there is an association between hip fractures and an increase in mortality. One year mortality rate after a hip fracture is estimated between 17 and 27 % [6–9].

The study has two primary hypotheses: first, that there are population factor variations in hip fracture incidence and, second, that there are systematic variations in mortality after hip fracture within the California population. A secondary hypothesis is that there has been a change in incidence trends over time from 2000 to 2011.

Several factors may be associated with hip fracture incidence such as age, gender, and race/ethnicity [1, 10]. California is a diverse state and provides an opportunity to examine these factors in relation to incidence of hip fracture and mortality following a hip fracture. The goal was to explore the effects of gender, age, and race/ethnicity, with regard to the incidence of hip fracture and 30-, 90-, and 365-day mortality rates in California from 2000 to 2009.

In the recent era of increasing health care quality measurement, these population data and analysis will be helpful in the interpretation of patient hip fracture incidence and mortality outcomes [11].

## Methods

This study was a population-based epidemiological review of all California Office of Statewide Health and Planning and Development (OSHPD) non-federal hospital admissions for hip fractures from 2000 to 2011.

Mortality data was extracted from the California State Death Statistical Master File (DSMF) records. Participants were assigned a linkage number, similar to a de-identified social security number, and data from initial hospital admission OSHPD records were linked to DSMF records, if applicable (e.g., if death had occurred). This linking method has previously been explained in detail [12].

**Fig. 1:**
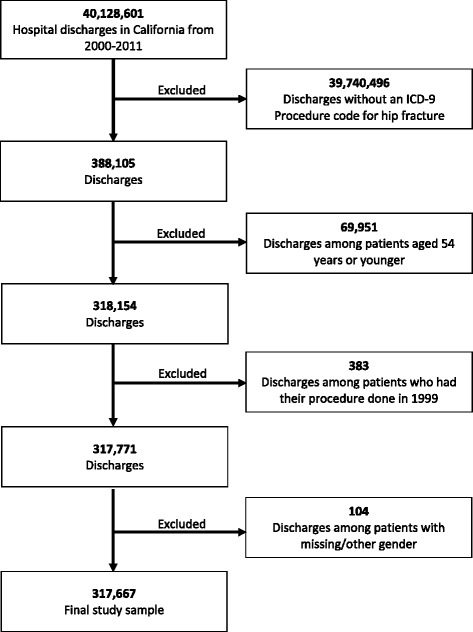
Flow diagram to show the creation of the study sample for the incidence of hip fractures

**Fig. 2:**
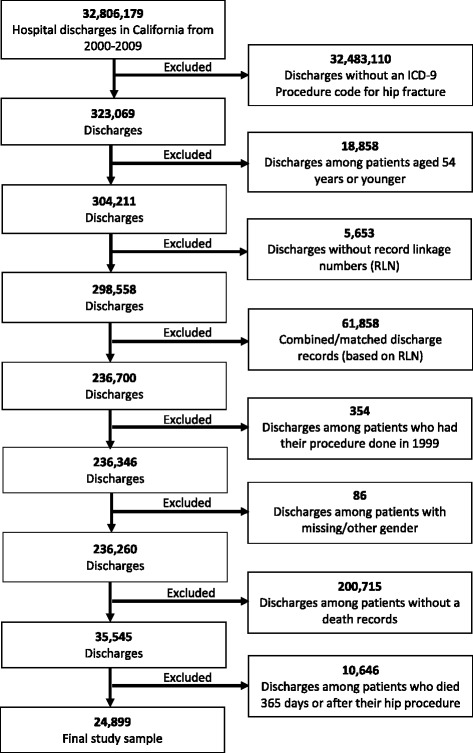
Flow diagram to show the creation of the study sample for mortality after hip fractures

Participants were any patient 55 years and older admitted with the primary International Classification of Disease, 9th Revision (ICD-9) procedure code for the treatment of hip fractures. These include partial hip arthroplasty (ICD9 81.52), internal fixation of bone without fracture reduction (ICD-9 78.55), closed reduction of fracture with internal fixation (ICD-9 79.15), open reduction of fracture with internal fixation, femur (ICD-9 79.35), and open reduction of separated epiphysis (ICD-9 79.55); see comments in study limitation section with regard to (ICD-9 79.55). There were a total of 317,677 hospital admissions for hip fractures over the 12-year span and 24,899 deaths following hip fractures (Figs. 1 and 2). All participants without linkage (substituted for social security) numbers were excluded from mortality rate calculations.

To evaluate the differences in the incidence of hip fracture, the outcome variable was incidence rate. To evaluate the difference in mortality, the outcome variables were 30-, 90-, and 365-day mortality. Hip fracture incidence rates were calculated based on hospital admission; a patient with multiple hip fractures could be counted twice toward hip fracture incidence rates.

Both incidences of hip fracture and mortality were evaluated by gender, age, race/ethnicity, and geographical area. Gender had two levels, male and female. Females were used as a reference group. Age was categorized in seven levels: 55–59, 60–64, 65–69, 70–74, 75–79, 80–84, and 85+. The 65–69 age group was used as the reference group because this is the first group that is eligible for Medicare. Race/ethnicity had six levels: Caucasian, African American, Hispanic, Asian, Native American, and other. Caucasians were used as a reference group because they were the largest racial/ethnic subgroup (Table 1).

**Table 1:** Demographics

Demographic variable	*N* (%)
Gender	
• Female	229,212 (72)
• Male	88,465 (28)
Age	
• 55–59	11,367 (3.6)
• 60–64	14,329 (4.5)
• 65–69	18,907 (6.0)
•70–74	28,717 (9.0)
• 75–79	48,269 (15.2)
• 80–84	70,353 (22.1)
• 85+	125,735 (39.6)
Race/ethnicity	
• Caucasian	250,407 (78.8)
• Hispanic	31,154 (9.8)
• African American	8818 (2.8)
• Native American	473 (0.1)
• Asian	16,811 (5.3)
• Other	4900 (1.5)

Rates were calculated by first calculating the count of hip fracture incidence in a relevant category. All analyses were weighted based on California population statistics, as described by the 2000 and 2010 US Census reports [13, 14]. For all years between 2000 and 2010, census distributions were interpolated. In addition, all age rates were weighted by the proportion of each relevant age group in the population. This prevented rates from being inflated by high rates in groups with low absolute counts (e.g., the oldest age groups). Differences in the incidence and mortality rate of hip fractures, based on various subgroups, were evaluated using Poisson regression models. Odds ratios (OR) are provided with 95 % confidence intervals (CI). All analyses were conducted in SPSS version 22 (Armonk, NY), Microsoft Excel (Redmond, WA), and R (Vienna, Austria).

Missing data were minimal for all analyses; cases with missing data were excluded from relevant analyses. Less than 3 % of the sample were missing a social security number and had to be excluded from death rate calculations. All participants without gender information were excluded as all analyses were split by gender. All participants with gender information also had age information. In addition, 1.6 % of people had missing race/ethnicity data and were excluded from those analyses.

## Results

### Hip fracture incidence

**Fig. 3:**
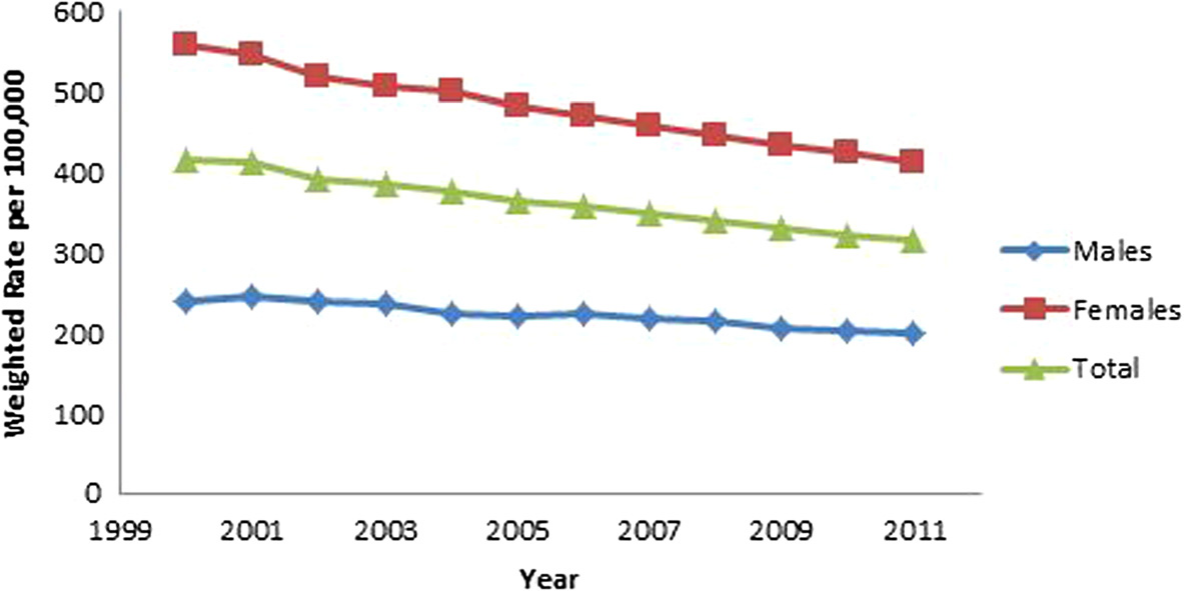
Hip fracture incidence rates over time

**Fig. 4:**
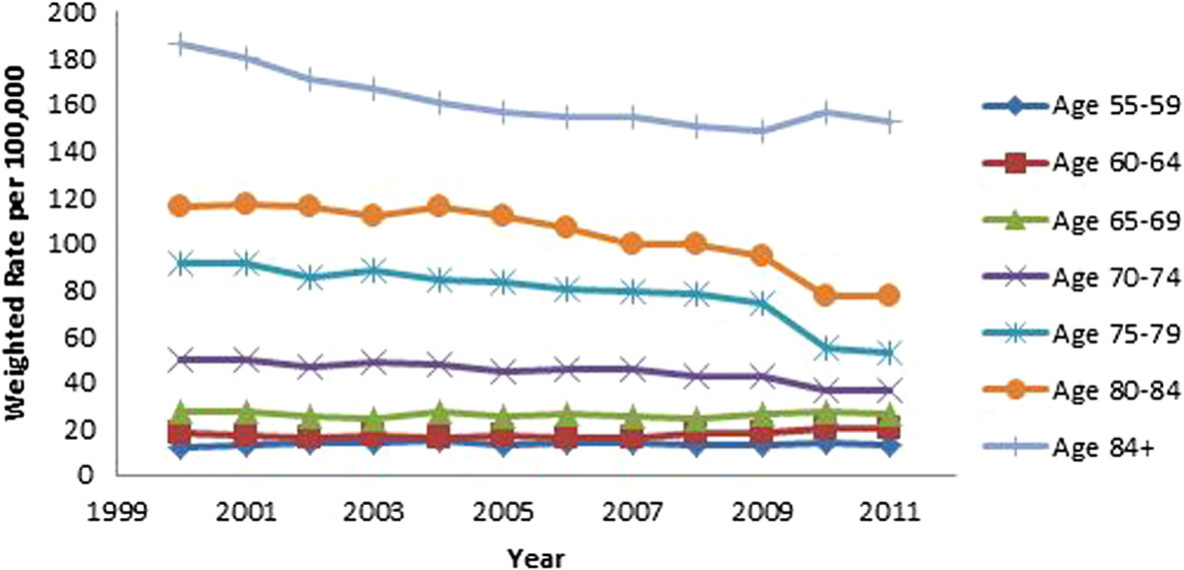
Female hip fracture incidence rates over time, by age group

**Fig. 5:**
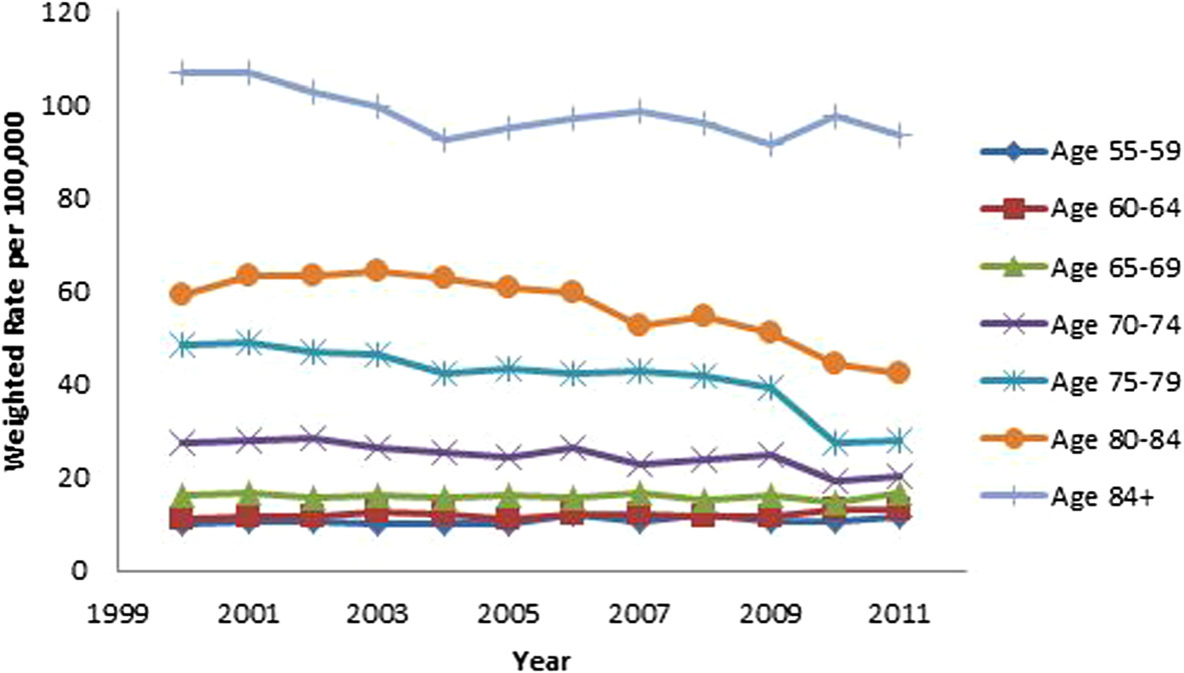
Male hip fracture incidence rates over time, by age group

**Fig. 6:**
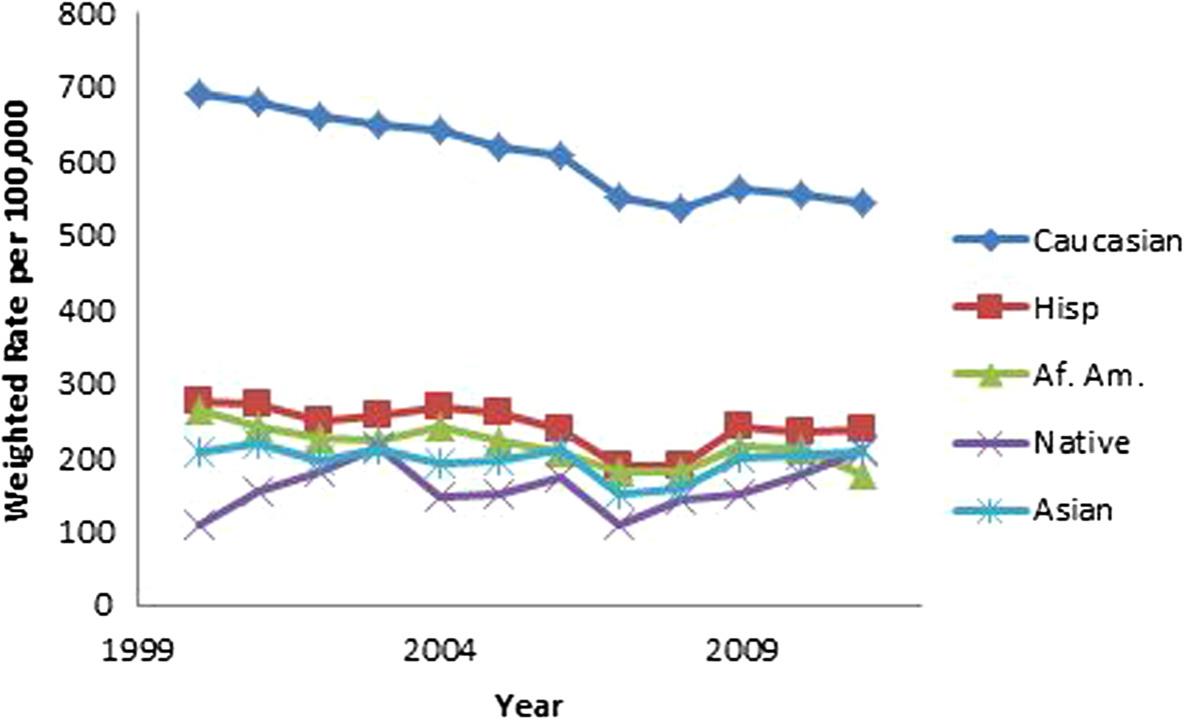
Female hip fracture incidence rates over time, by race/ethnicity

**Fig. 7:**
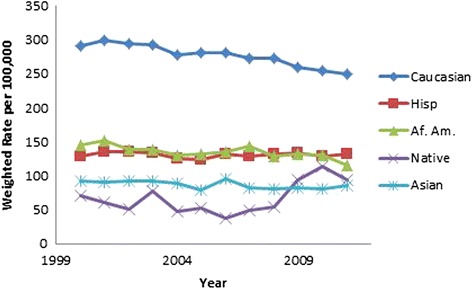
Male hip fracture incidence rates over time, by race/ethnicity

All results were weighted using population rates from the US Census Bureau [13, 14] and are presented in Table 2. Hip fracture rates decreased over time (OR 0.98, 95 % CI 0.98, 0.98, *p* < .001, Fig. 3). Males were found to have a lower incidence of hip fractures than females (OR 0.46, 95 % CI 0.46, 0.47, *p* < .001, Fig. 3). As gender differences were dramatic, all subsequent analyses were performed on males and females separately. As a person ages, they are more likely to sustain a hip fracture; females 85 years and above were 18.73 times more likely to sustain a hip fracture than those aged 65–69 (95 % CI 18.36, 19.10, *p* < .001). The relationship in males was even more dramatic, those 85 and above were 32.79 times more likely to sustain a hip fracture than the reference group (95 % CI 32.15, 33.43, *p* < .001; (Figs. 4 and 5). Caucasians had the highest incidence of hip fracture across all race/ethnicity groups; Native Americans had the lowest rates in reference to Caucasians (females OR 0.26, 95 % CI 0.24, 0.29, *p* < .001; males OR, 0.26, 95 % CI 0.21, 0.30, *p* < .001, Figs. 6 and 7).

**Table 2:** Adjusted odds ratio and 95 % confidence intervals (95 % CI) of gender, age group, and race/ethnicity in hip fracture patients from 2000–2011

Variable		
Year	0.98 [0.98, 0.98]^a^	
	Female	Male
Gender	1.00	0.46 [0.98, 0.98]^a^
Age group (years)		
55–59	0.64 [0.61, 0.66]^a^	0.66 [0.64, 0.69]^a^
60–64	0.61 [0.60, 0.62]^a^	0.66 [0.64, 0.69]^a^
65–69	1.00	1.00
70–74	1.92 [1.88, 1.95]^a^	1.92 [1.84, 1.99]^a^
74–79	4.06 [3.98, 4.14]^a^	4.62 [4.53, 4.71]^a^
80–84	10.70 [10.49, 10.91]^a^	12.18 [11.95, 12.42]^a^
85+	18.73 [18.36, 19.10]^a^	32.79 [32.15, 33.43]^a^
Race/ethnicity		
Caucasian	1.00	1.00
Asian	0.32 [0.32, 0.33]^a^	0.32 [0.30, 0.33]^a^
African American	0.35 [0.35, 0.36]^a^	0.49 [0.47, 0.51]^a^
Hispanic	0.39 [0.39, 0.40]^a^	0.48 [0.0, 0.04]^a^
Native American	0.26 [0.24, 0.29]^a^	0.25 [0.21, 0.30]^a^

^a^ *p* < 0.001

### Hip fracture mortality

All results were weighted using population rates from the US Census Bureau [13, 14]. Thirty, 90 and 365 day mortality results presented in Table 3. Over time, mortality rates decreased in both genders at 30-day post procedure (OR 0.97, 95 % CI 0.96, 0.98, *p* < .001, Fig. 6), 90-day post procedure (OR 0.97, 95 % CI 0.96, 0.98, *p* < .001), and 365-day post procedure (OR 0.98, 95 % CI 0.98, 0.98, *p* < .001, Fig. 6). Males were nearly two times more likely to die within 30 days of a procedure than females (OR 1.79, 95 % CI 1.72, 1.86, *p* < .001) and were more likely to die at all assessed time points. Across all time mortality rate assessments, there were no differences in mortality rates for males between age groups 55–59, 60–64, and 64–69; however, females aged 55–59 had lower mortality rates than those aged 64–69 (OR 0.73, 95 % CI 0.55, 0.98, *p* < .05). For both genders, beyond age 69, risk of death increased as a person aged. For both genders, Caucasians were more likely to die at 30, 90, and 365 days than all other races. Asians had the lowest mortality rates as compared to Caucasians (female, 30-day OR 0.59, 95 % CI 0.51, 0.68, *p* < .001; male, 30-day OR, 0.62, 95 % CI 0.52, 0.74, *p* < .001).

**Table 3:** Adjusted odds ratio and 95 % confidence intervals (95 % CI) of gender, age group, and race/ethnicity in hip fracture patients from 2000–2009

	30-day mortality		90-day mortality		365-day mortality	
Variable						
Year	0.97 [0.96, 0.98]^a^		0.97 [0.96, 0.98]^a^		0.98 [0.98, 0.98]^a^	
	Female	Male	Female	Male	Female	Male
Gender	1.00	1.79 [1.72, 1.86]^a^	1.00	1.63 [1.57, 1.70]^a^	1.00	1.43 [1.41, 1.46]^a^
Age group (years)						
55–59	0.73 [0.55, 0.98]^c^	0.79 [0.60, 1.03]	0.84 [0.67, 1.04]	0.70 [0.56, 0.89]^b^	0.96 [0.81, 1.15]	0.74 [0.61, 0.90]^b^
60–64	0.87 [0.67, 1.21]	0.95 [0.74, 1.23]	0.89 [0.73, 1.08]	0.98 [0.81, 1.19]	0.99 [0.85, 1.16]	0.99 [0.83, 1.18]
65–69	1.00	1.00	1.00	1.00	1.00	1.00
70–74	1.18 [0.97, 1.44]	1.60 [1.29, 1.98]^a^	1.20 [1.02, 1.40]^c^	1.45 [1.21, 1.72]^a^	1.30 [1.15, 1.46]^a^	1.39 [1.21, 1.60]^a^
75–79	1.49 [1.25, 1.78]^a^	2.20 [1.81, 2.68]^a^	1.43 [1.25, 1.64]^a^	1.99 [1.70, 2.33]^a^	1.54 [1.37, 1.73]^a^	1.86 [1.62, 2.13]^a^
80–84	1.73 [1.48, 2.02]^a^	2.61 [2.18, 3.11]^a^	1.80 [1.57, 2.07]^a^	2.41 [2.10, 2.77]^a^	1.95 [1.74, 2.20]^a^	2.32 [2.06, 2.61]^a^
85+	3.25 [2.78, 3.81]^a^	4.22 [3.54, 5.03]^a^	3.32 [2.95, 3.73]^a^	3.71 [3.23, 4.25]^a^	3.56 [3.23, 3.93]^a^	3.42 [3.04, 3.85]^a^
Race/ethnicity						
Caucasian	1.00	1.00	1.00	1.00	1.00	1.00
Asian	0.59 [0.51, 0.68]^a^	0.62 [0.52, 0.74]^a^	0.52 [0.46, 0.58]^a^	0.59 [0.51, 0.68]^a^	0.51 [0.46, 0.56]^a^	0.57 [0.51, 0.64]^a^
African American	0.78 [0.67, 0.91]^c^	0.64 [0.53, 0.78]^a^	0.78 [0.68, 0.89]^a^	0.66 [0.56, 0.79]^a^	0.73 [0.65, 0.82]^a^	0.73 [0.63, 0.83]^a^
Hispanic	0.79 [0.71, 0.87]^a^	0.68 [0.61, 0.77]^a^	0.71 [0.66, 0.77]^a^	0.66 [0.60, 0.72]^a^	0.66 [0.63, 0.70]^a^	0.65 [0.60, 0.70]^a^

^a^ *p* < 0.001; ^b^ *p* < 0.01; ^c^ *p* < 0.05

## Discussion

### Discussion of hip fracture incidence

There are multiple individual risks for the occurrence of a hip fracture including but not limited to osteoporosis, smoking, general health status, medical co-morbidities, exercise, and socio-economic status [1, 15]. In this study, we found that Caucasian females, aged 85+ were at the most risk for a hip fracture. Figure 3 (procedure rates over time) graphically illustrates the significant difference in hip fracture incidence with men having an incidence about half that of women. This study reports results consistent with similar studies done with Medicare data, European population studies, and previous studies on California data[1, 4, 16, 17]. When evaluating programs designed to reduce the incidence of hip fractures the decreasing incidence must be factored into any program evaluation [16].

Figures 4 (female hip fracture rates over time) and 5 (male hip fracture rates over time) graphically illustrates that hip fracture incidence for men and women over the age of 70 declined over the study time period. These declines are an extension of the trend noted by Brauer et al. [1]. They suggest an improved trend for the overall California population in line with the Swiss population [18]. This improved trend may be partially explained by widespread prescription of bisphosphonate medications but improved population health, decreasing incidence of tobacco use and public health promotion of increased activity and healthy lifestyles are also possible contributors to this trend [9, 19, 20]. Kannus et al. [21] in agreement with this statement speculate that the biological basis for this declining rate is multifactorial.

Figures 6 (female hip fracture rates over time, by race/ethnicity) and 7 (male hip fracture rates over time, by race/ethnicity) graphically illustrate that white men and women have a significantly higher incidence of hip fractures compared to the remainder of the population. The decline in incidence rate over the study time period has been more significant for white men and women.

Figure 6 (female hip fracture rates over time, by race/ethnicity) is an interesting and consistent extension of Fig. 1a published in Zingmond et al. [8] for the period 1983–1998. The ethnic disparities are consistent with the data of Silverman and Madison from 1983–1984 [5]. When compared to Kanis et al., the data of the white population has an incidence rate similar to Sweden, Norway, Austria, and Ireland [22]. The remainder of the population has an incidence rate consistent with Spain, Mexico, and Chile, which are similar to a review done by Cheng et al. [23]. Wright et al. in a Medicare sample population found in a similar time period that incidence of Hispanic hip fractures had not declined, similar to our study [2]. Investigations of the race/ethnicity factor for hip fractures has been further confused by the changing demographics of the US population.

### Discussion of hip fracture mortality

There are multiple individual risks for the occurrence of mortality following a hip fracture including but not limited to body pre-operative functional status, pre-operative cognitive status, congestive heart failure, general health status, diabetes, other medical co-morbidities, and occurrence of post-operative patient complications (notably sepsis) [24, 25]. In this study, we found that Caucasian males aged 85+ was the profile for patients at the most risk for a hip fracture mortality. That males were more likely than their female counterparts to die following a hip fracture was particularly interesting because females had the highest hip fracture incidence rates. The increase in mortality risk associated with increasing age and male gender has been widely noted in other studies of this topic [7, 26–28].

**Fig. 8:**
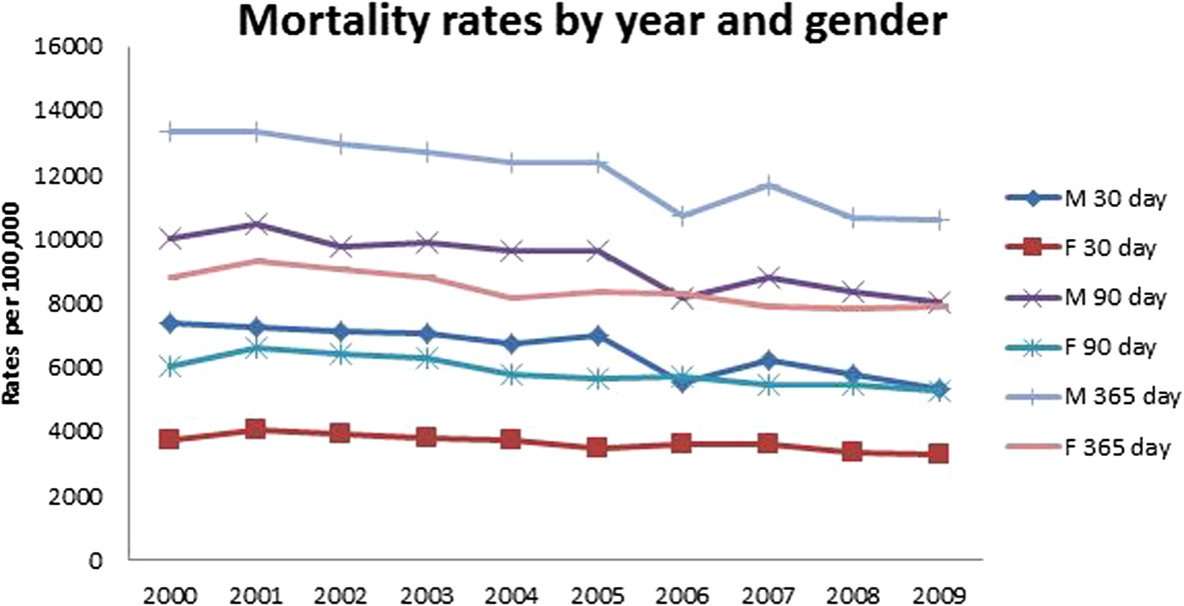
30-, 90-, and 365-day mortality rates over time

Figure 8 (30-, 90-, and 365-day mortality rates over time) graphically illustrates that all mortality rates decreased over the study time period. This is a resumption of a trend to decreased mortality that Brauer et al. noted had stalled in about 1998 [1].

**Fig. 9:**
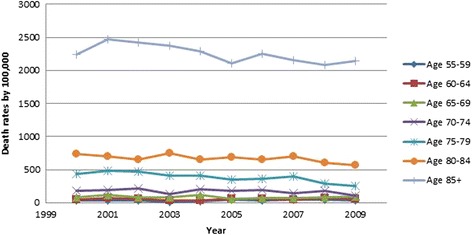
Female 30-day mortality rates over time, by age

**Fig. 10:**
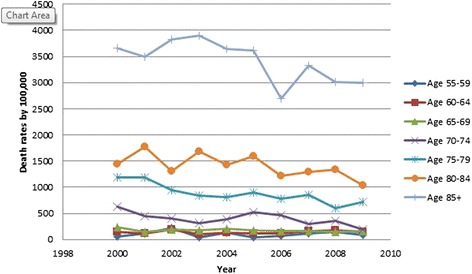
Male 30-day mortality rates over time, by age

Figures 9 and 10 graphically illustrate that the incidence of 30-day mortality varies by decade of life for both men and women. These gender- and age-specific baseline mortality rates will be helpful to risk adjust the incidence of mortality after hip fracture care. Mortality rates are being introduced in the USA and elsewhere as a quality measure for hip fracture care [11]. There has been an increase in the use of systematic interventions including co-operative care between surgeons and medical practitioners, attention to pain management, delirium prevention, early surgery, and aggressive mobilization [29–32].

Penrod et al. in a study of approximately 3000 patients from 1997–1999 found that white patients enjoyed a mortality risk advantage compared to the rest of the study population [25]. Our study was based on a much larger and more comprehensive study population. Sterling documented a gap in the literature with regard to racial and ethnic differences in the survival of US hip fracture patients [33]. A more current literature search has failed to find current studies on this topic. One older citation was based on a very different racial/ethnic population compared to 2009 [3].

### Limitations

One limitation was that we selected our dataset based on the principal procedure code only. Other procedure codes are recorded, and so there is a possibility that we missed some patients who had a hip fracture that was not coded as their principal procedure. This is ICD-9 data and laterality is not a data element.

Although the patient data record contains information regarding the hospital where the surgery occurred, there is no data with regard to transfers of patients for admission, subsequent re-operations at a second hospital for the same fracture, nor data with regard to the individual attending surgeon. These factors would all be useful information for the analysis [34, 35].

Another limitation is the inadvertent inclusion of a small group of patients (*n* = 296) who had a principal procedure code of 79.55 (open reduction of separated epiphysis, femur) and were older than age 55. Since this is a pediatric orthopedic procedure, this combination of principal procedure code and age is likely the result of incorrect coding at the hospital level. However, it is unknown if the incorrect coding was in the age of the patient or the principal procedure code. The number of patients is small (0.09 %), data analysis was not re-done, and inclusion of this subset should not significantly affect the results.

The last important limitation of using the OSHPD dataset was the lack of important clinical risk data (e.g., smoking status, substance abuse status, socio-economic status information, opioid use, pre-operative mobility, and cognitive status, etc.) [1, 15]. Due to the nature of the dataset, there was no way to analyze any of these factors and therefore we must accept this limitation of applying these results to health effectiveness research.

## Conclusion

This California state-wide population-based study of a large and diverse population shows a significant reduction in hip fracture incidence over the study period of 2000–2011 and a corresponding reduction in mortality over the study period of 2000–2009. There are significant gender, age, race/ethnicity disparities for both hip fracture incidence and mortality in subpopulations that will allow for targeted population interventions and opportunities for further research. Further, these data will provide baseline information to assess and risk stratify outcomes and interventions.

### Ethics approval

IRB approval was received from Community Medical Center (#2012094) and the State of California’s Committee for the Protection of Human Subjects (#12-10-0840).
